# Visual rehabilitation of patients with corneal diseases

**DOI:** 10.1186/s12886-020-01436-7

**Published:** 2020-05-06

**Authors:** Michael Oeverhaus, Dirk Dekowski, Herbert Hirche, Joachim Esser, Barbara Schaperdoth-Gerlings, Anja Eckstein

**Affiliations:** 1 Department of Ophthalmology, University Hospital Essen, Hufelandstr. 55, 45147 Essen, Germany; 2 Institute of Medical Informatics, Biometry and Epidemiology, University of Duisburg-Essen, Essen, Germany

**Keywords:** Low vision, Low vision aids, LVAs, Magnifier, Corneal diseases, Corneal haze, Corneal densitometry

## Abstract

**Background:**

Although most patients with visual impairment due to corneal diseases can be treated successfully with surgery, some require visual rehabilitation to restore reading ability. To evaluate the best LVAs especially in terms of reading speed and characterize this specific patient group we performed a prospective, randomized cross-over trial.

**Methods:**

All 34 patients underwent a detailed examination (slit-lamp, funduscopy, SD-OCT, ETDRS) as screening. Only patients with corneal diseases without other ocular diseases were included. Reading-speed was assessed with International-Reading-Speed-Texts (IReST) consecutively with five different LVAs (low vision aids) during one day in a randomized cross-over design. Corneal haze was quantified with corneal densitometry (Pentacam).

**Results:**

Patients were either visually impaired (*n* = 28), severely impaired (*n* = 4) or legally blind (*n* = 2). Patients read significantly faster with LVAs (*p* < 0.0001). Fastest reading speed could be achieved with video magnifier (CCTV). Optical magnifier and portable-electronic magnifier enabled significantly lower reading speeds (*p* < 0.01). In a subgroup of patients (VA < 3/60,*n* = 6) black background enabled patients to read significantly faster compared to white background (*p* = 0.03).

**Conclusion:**

Patients with low magnification requirement can be treated successfully with optical LVAs and portable-electronic magnifiers. More severely afflicted patients need a CCTV. Black background enables fastest reading-speeds, probably due to less blinding. Visual impairment can be estimated with corneal densitometry. Our trial confirms the capability of LVAs to successfully restore the reading ability in patients with corneal diseases, which is a crucial part of visual rehabilitation.

**Trial registration:**

This trial was registered at the German Clinical Trials Register as DRKS00010887 at 09.08.2016.

## Background

Visual impairment (VI) is one of the most challenging disabilities worldwide. An increasing number of people are at risk for VI caused by chronic eye diseases due to the globally growing elderly population [1]. Therefore, the requirement for visual rehabilitation is estimated to increase in the near future [2]. In Germany, there are estimated to be 1.1 million visual impaired persons (Visual acuity, VA < 6/18), in addition to 160,000 legally blind people (WHO Grade 4, VA ≤1/60) [3, 4]. The most common causes for VI in Germany are age-related macular degeneration (AMD), glaucoma and diabetic retinopathy [2]. A smaller group of patients suffers from VI due to corneal diseases, like corneal opacities caused by thermal/chemical burns, corneal dystrophies and ocular graft versus host disease (GvHD), as well as keratoconus. Most cases can be treated successfully with surgical methods like perforating keratoplasty (PK), descemet membrane endothelial keratoplasty (DMEK), deep anterior lamellar keratoplasty (DALK), amnion membrane transplantation and limbal stem cell transplantation. However, due to ocular risk factors, like vascularization, uncontrolled intraocular pressure (IOP) and uveitis some patients cannot be treated successfully with these procedures [5]. In addition, some patients cannot undergo surgery due to comorbidities, like heart diseases or refuse surgery as a result of their age or fear. Even patients who can be treated with surgery often wait months to years for a graft, depending on the procedure. Patients with keratoconus can usually be treated with contact lenses, but some patients suffer from pain while wearing and cannot endure this treatment. Besides these reasons, insufficient health insurance can also keep patients from optimal medical care. All these patients have to endure VI and its consequences, which comprise, besides reading disability, problems in performing tasks of daily living and social interactions. These problems lead to a decreased self-sufficiency and more dependency on relatives and caring persons. Due to these detrimental consequences several studies were able to show, that the quality of life of visual impaired patients is drastically decreasing and there is a higher prevalence of depression [6–8]. Furthermore, patients have more accidents and falls, which leads to a higher morbidity and mortality [9]. Patients with VI should hence undergo visual rehabilitation. By making best use of the remaining vision, rehabilitation aims to improve mobility, reading ability, and consequently autonomy of patients. By low vision aids (LVAs), like optical magnifiers, electronic desk video magnifiers (closed-circuit television, CCTV) and portable electronic vision enhancement systems (p-EVES) reading ability can be improved or restored and consecutively quality of life as well [10–15]. The adaption of the suitable LVA for each patient is depending on the disease, the magnification requirement, former reading behavior and other individual factors [16, 17]. Furthermore, the best LVA is highly depending on the task it will be used for. Since electronic LVAs are much more expensive compared to optical LVAs, visual rehabilitation should incorporate this as well [18]. Therefore, the visual rehabilitation process is very complex and time consuming [19]. Due to glare sensitivity and other concomitant problems, like dry eye, adaption of LVAs is especially difficult in patients with corneal diseases. Although it is known that the underlying disease is important for visual rehabilitation there is no published data regarding these patients [16]. This probably results from the rareness of irreversible VI due to corneal diseases. Most studies rather focus on the main causes for visual impairment, like AMD [19, 20]. The best contrast settings have also only been evaluated for retinal diseases [21–23]. To evaluate LVAs, it is a necessity to measure the reading speed of the patients to show that reading performance can be improved and quantify this improvement, according to a Cochrane Review [24]. This can be done by single sentences charts (Radner, MNREAD) or paragraphs, like the International reading speed texts (IReST). We chose IReST to measure reading speed, because it represents leisure reading (books, newspaper) and provides standardized paragraphs matched for linguistic difficulty to assess reading speed in repeated measurements [10, 25, 26]. Apart from that, it is recommend that patients’ preference and characteristics should be assessed [24]. Therefore, in this prospective, randomized cross-over trial, we aimed to characterize patients with visual impairment due to corneal diseases and evaluate the best low vision aid for this group in terms of objective reading performance and patient-reported rating.

## Methods

### Study population

Patients for this trial were recruited from our low vision and corneal diseases referral center between July 2016 and November 2017. Patients meeting the following inclusion and exclusion criteria were included (*n* = 34): visual impairment (BCVA < 6/18) caused by a corneal disease (e.g. corneal dystrophies, thermal/ chemical burn, ocular GVHD, keratoconus), no visual limitation due to other concomitant ophthalmic diseases (e.g. AMD, glaucoma etc.), ability to converse, read and write German fluently, mentally competent, no diagnosed depression, no physical disability that prevents to operate LVAs [27]. The recruitment and research protocols were reviewed and approved by the Institutional ethics commission, and written informed consent was obtained from all study participants in compliance with the Declaration of Helsinki. The trial was registered with the German Clinical Trials Register (DRKS00010887).

### Morphological examination

All patients were subjected to a standardized ophthalmological examination of both eyes including slit-lamp biomicroscopy and funduscopy. Intraocular pressure was assessed with Goldmann Applanation Tonometry (GAT) and the corneal surface examined with fluorescein staining. Furthermore, all subjects underwent slit-lamp photography, laser interference visual acuity measurement (LIVA) and corneal biometry (Pentacam HR, Oculus GmbH, Wetzlar, Germany) of both eyes. To ensure that patients had no concomitant retinal diseases we performed funduscopy and SD-OCT (Spectral-Domain Optical Coherence Tomography [Spectralis HRA + OCT, Heidelberg Engineering, Germany]) in all patients. To characterize corneal status, corneal haze was examined quantitatively with the densitometry program of the Pentacam. Here a rotating Scheimpflug camera is combined with a static camera to acquire multiple photographs of the anterior eye segment. We used the 25-scans setting in the automatic release mode to minimize examiner-induced errors. A software module enables a standardized corneal densitometry analysis [28, 29]. It measures corneal backscattered light over a 12-mm-diameter area and full corneal thickness. Corneal densitometry can also be measured in four annular zones centered on the apex of the cornea (0–2, 2–6, 6–10, and 10–12 mm in diameter). The densitometry measurement can also be provided for the anterior layer (first 120 mm), central layer (from the first 120 mm to the posterior 60 mm), and the posterior layer of the cornea (60 mm). Densitometry is expressed in grayscale units (GSU), ranging from a minimum light scatter of 0 (no corneal haze) to a maximum light scatter of 100 (totally opaque cornea) [29, 30]. Corneal densitometry has previously been established to quantify the manifestation of several corneal diseases and was therefore chosen to characterize this patient group [31–35]. Slit lamp and Pentacam examinations were all performed by one ophthalmologist to secure homogeneity and reproducibility. Only Pentacam images with good quality were included, according to the integrated quality assurance software.

### Functional examination

Following enrollment, patients underwent a visual assessment to describe multiple aspects of visual function. Distance visual acuity was determined in a standardized manner for each eye (right eye first) according to the ETDRS (Early Treatment of Diabetic Retinopathy Study) protocol. All measurements were performed in the same room under the same conditions (dark room). Internally illuminated Bailey-Lovie charts (Lighthouse International, NY, USA) were used to determine the monocular best corrected visual acuity at 2 m distance. Letter by letter scoring was employed in accordance with the method described by Ferris [36]. Refraction was performed at 2 m with Chart “R” according to the EDTRS protocol. Briefly, the most positive or least negative spherical and least negative cylindrical lens consistent with best visual acuity was used. In addition, near VA was assessed binocularly at 40 cm using Bailey-Lovie charts and best correction for the near distance [37]. The results are presented in logMAR units. According to the WHO definitions patients were categorized into different classes of visual impairment: WHO grade 1 (VA < 6/18), WHO Grade 2 (VA < 6/60), WHO Grade 3 (< 3/60) and Blindness (WHO Grade 4, ≤1/60). For statistical analysis we further grouped Grade 1–2 as moderate VI and 3–4 as severe VI. The appropriate magnification was assessed as in clinical routine using standardized charts with sentences in different print sizes at 25 cm distance under standardized illumination with best correction for near distance, described by others before [19, 38]. Briefly, the smallest print size which still enabled fluent reading was chosen as required magnification which indicates by how much newspaper print size has to be magnified. The LVAs already owned and used regularly by the patients were assessed with a questionnaire as well as their glare sensitivity.

### Low vision aids

We used the International Reading Speed Texts (IReST) to assess the reading speed of patients. To be comparable between each other, the paragraphs were originally designed by linguists who matched content, length (paragraphs, on average 132 words), difficulty (reading ages 10–12 years) and linguistic complexity. All paragraphs are printed in Times New Roman 10 point with inter-line and letter spacing similar to newspaper print. Reading speed (right words per minute, wpm) was first assessed with best correction following the protocol described prior to this [39]. In short, subjects had to read the text aloud as quickly as possible without corrections. The wrongly read words were counted and subtracted from the total number of words of the paragraph. Time was measured beginning at the uncovering of the text to have an exact starting point. After this baseline reading speed, 5 LVAs were tested using different paragraphs (No. 2,4,5,6,8) adapted to the magnification requirement of the patient: optical magnifier (Schweizer Optik), p-EVES (Reinecker *Mano M,* 4.3″ display), CCTV (Reinecker *Veo*, 22″ display) black font on white background (normal contrast), white-on-black (reversed polarity) and green-on-black. The order of testing was randomized by block randomization to minimize confounding factors. Between testing the different LVAs, all patients had to rest to limit exhaustion and apply artificial tears if required. If the patient was not used to any of the devices, a training session was conducted before the reading test, followed by a break. Patients were trained how to handle the device and had to read, using it to familiarize the patient with the LVA. After completing all reading tests, patients were asked to rate the LVAs from 0 to 10 (worst - best). Reading speed was measured as right words per minute and compared to the normative reading rates of each text, provided by the IReST Study group [25, 26]. To compare the different LVA, results are presented as percentage of normal reading speed **(**$\frac{\boldsymbol{wpm}}{\boldsymbol{nwpm}}$**).** Therefore, small differences of normal reading speed between texts are irrelevant in the statistical comparison of the LVAs. This was necessary since IReST offers texts with the same performance level, but not five with identical level (max. 4). Still, the maximum difference between the used texts was only nine words. To also enable comparison with other studies reading speed is also presented as right words per minute.

### Statistical evaluation

For metric data, median values and range or the mean and standard deviation (SD±) were calculated and differences between groups were evaluated with Student’s t-test (two-tailed), if D’Agostino-Pearson normality-test showed normal distribution, if not with Mann-Whitney-Test or Wilcoxon signed rank test. Fisher’s exact test was used to evaluate group distributions of binary variables. Repeated measures analysis of variance (ANOVA) was performed to examine the effect of LVA type on reading speed. For exploratory investigation of the influence of baseline visual acuity, reading speed was also analyzed for two groups of participants (VA above and below 20/400). Linear regressions have been performed to test the association between functional and morphological parameters. Multiple linear regression has been performed to assess further influential factors. Level of statistical significance was defined two-tailed as 2α < 0.05. *P*-values are given with α-adjustment for multiple testing (Bonferroni). All calculations were performed with SPSS (IBM SPSS Statistics, Chicago, IL, USA, Version 22.0.0,) and Graph Pad Prism (Prism 6 for Windows, Software Inc., San Diego, CA, USA, Version 6.01).

## Results

### Study population

Recruitment took place between 1st July 2016 and 31st November 2017. A total of 46 consecutive patients with corneal diseases were recruited by ophthalmologists. Twelve patients were ineligible because of concomitant retinal diseases, resulting in 34 subjects (23–86 years) participating in this clinical trial. A breakdown of demographic characteristics of these participants is presented in Table 1. Most patients were resident with their family or partner (65%), retired (56%) and had completed a vocational training (50%). Visual impairment (WHO grade 1) was most common (82%), whereas severe impairment (Grade 3) and blindness (Grade 4) were less frequent. Visual characteristics resembled this classification (see Table 2). Most patients suffered from severe glare sensitivity (65%).

**Table 1:** Demographic characteristics of participants recruited to the study

	*n*	
Age (years)	34	65.7 ± 14.2 [23,86]
Females/ Males	17/ 17	50% /50%
Duration of disease (years)	34	10 [1–76]
Visual impairment status		
Visually impaired (≤20/63)	28	82.3%
Severely impaired (≤20/400)	4	11.8%
Blind (≤20/1000)	2	5.9%
Residential situation		
Alone	12	35.3%
With spouse/ partner/family	22	64.7%
Employment status		
Employed	9	26.5%
Unemployed	6	17.6%
Retired	19	55.9%
Education and professional training		
Secondary school	6	17.6%
Completed vocational training	17	50%
Qualification for university	8	23.5%
University degree	3	8.8%

Unless otherwise stated data are means ±SD or proportions (%) or median [range]

**Table 2:** Visual characteristics and symptoms

BCVA (better eye, logMAR)	0.70 [2–0.4]
BCVA (other eye, logMAR)	1.1 [n.l.-0.6]
Binocular near VA	0.63 ± 0.2
Corneal haze (GSU)	35.7 ± 14
Magnification need	5 [1.6–30]
Glare sensitivity (n)	
Low	26.5% [8]
Moderate	11.8% [4]
Severe	61.8% [20]

Unless otherwise stated data are means ±SD or proportions (%) or median [range]

### Ophthalmological examinations

**Fig. 1:**
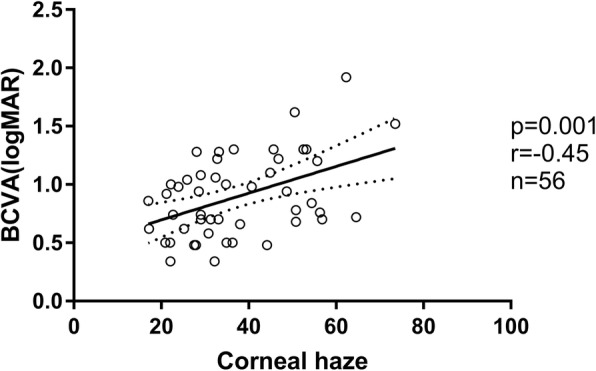
Linear Regression showed a significant correlation between the degree of corneal haze (0–100 Units, no haze – total opacification) in the central annular zone (0-6 mm) and best corrected visual acuity (BCVA)

All 34 patients included in the analysis met the inclusion and exclusion criteria and were visually impaired due to a corneal disease (s. Table 3 for primary diagnosis). SD-OCT scans could not be performed in eight patients due to high grade corneal haze. With the help of funduscopy and LIVA it was assured that patients had no concomitant disease which caused visual impairment. Pentacam scans could be performed in 63 eyes. Corneal haze was too dense in 9 patients. In six cases, Pentacam scans had to be dismissed because the quality standards could not be achieved. Thus, 56 eyes (75.7%) were included in the final analysis. The measurements of all layers for the central annular zone (0-6 mm) were considered to be most important for visual acuity and hence used for further analysis. A linear regression was performed to test if the quantified corneal haze correlates with visual acuity. The model showed a significant correlation (F1,55 = 11.8; *p* = 0.001, *r* = − 0.45, s. Fig. 1): A higher degree of corneal haze resulted in decreased visual acuity. To explore the unexplained variability, we performed a multiple linear regression and added sequentially age and LIVA. This model, in which corneal haze remained the most important independent variable, could predict a greater percentage of the variability (F2,55 = 10.1; *p* < 0.0001, *r* = 0.41), but showed still a wide range.

**Table 3:** Primary ocular pathologies among the participants (*n* = 34)

Primary diagnosis	
Thermal/chemical burn	7
Corneal dystrophy	6
Ulcer	6
Keratoconus	3
GvHD	3
Other	9

### Low vision aids

Most patients were adequately supplied with LVAs (79%). However, 10 patients had either no (*n* = 3) or insufficient LVAs (*n* = 7). Patients with moderate VI (WHO 1–2) were mostly aided by optical LVAs, whereas patients with severe VI (WHO 3–4) had significantly more often electronic LVAs (see Table 4).

**Table 4:** Low Vision aids of the patients grouped for grade of visual impairment

LVA	WHO Grade 1–2 (*n* = 28)	WHO Grade 3–4 (*n* = 6)	P
No LVAs	11% [3]	–	0.93^a^
Insufficient LVAs	18% [5]	33.3% [2]	0.21^a^
Optical LVAs	89% [24]	83.3% [5]	0.55^a^
Quantity per patient	1 [1,6]	1 [0,2]	0.46^b^
Electronic LVAs	25% [6]	88.3% [5]	**0.01**^**a**^
Quantity per patient	0 [0,3]	1 [0,3]	**0.04**^**b**^
CCTV	14% [4]	66.7% [4]	**0.02**^**a**^
p-EVES	14% [4]	50% [3]	0.09^a^
Consumer electronics	46% [12]	83.3% [5]	0.18^a^
Quantity per patient	0 [0,4]	1 [0,2]	0.34^b^
Smartphone	46% [12]	83.3% [5]	0.18^a^
Tablet	21% [40]	50% [3]	0.31^a^
Camera	14% [4]	–	0.77^a^
Cut-off filter glasses	25% [6]	16.7% [1]	0.66^a^

*CCTV* Closed-circuit television; *p-EVES* portable electronic vision enhancement systems

Unless otherwise stated data are proportions (%) or median [range]

a: Fishers exact test b: Mann-Whitney-test

Interestingly, half of the patients used consumer electronics like smartphones and tablets to magnify texts. As main reasons, they reported to use devices that are less stigmatizing and more convenient, since they are carried with anyway (especially smartphones).

Before testing the reading speed, the magnification requirement was assessed in all patients (s. Table 2) and LVAs adapted accordingly. The reading test was performed with five different LVAs in a randomized order to evaluate the LVA with the best reading speed (see Table 5 for reading speeds). Upfront, reading was assessed with best correction without any LVA. Only six patients (16%) could read at intervals of 33 cm, whereas all patients could read with appropriate LVAs. Reading with an optical LVA and p-EVES was impossible in seven and two patients, respectively. Whereas optical magnifier could enable in average fluent reading (80 wpm, [41]) for patients with a max. 5-fold magnification requirement, this was not possible for patients with ≥6-fold. They showed a significantly decreased reading speed (*p* < 0.0001).

**Table 5:** Reading speed (right words per minute, wpm) with different LVAs

Best correction	21.4 ± 46
CCTV	
Normal contrast	101.4 ± 43
Reversed polarity contract	102 ± 36
Green-on-black contrast	98.8 ± 35
p-EVES	69.8 ± 35
Optical LVA	65.1 ± 42

Unless otherwise stated data are means ±SD or proportions (%) or median [range]

*CCTV* Closed-circuit television; *p-EVES* portable electronic vision enhancement systems

**Fig. 2:**
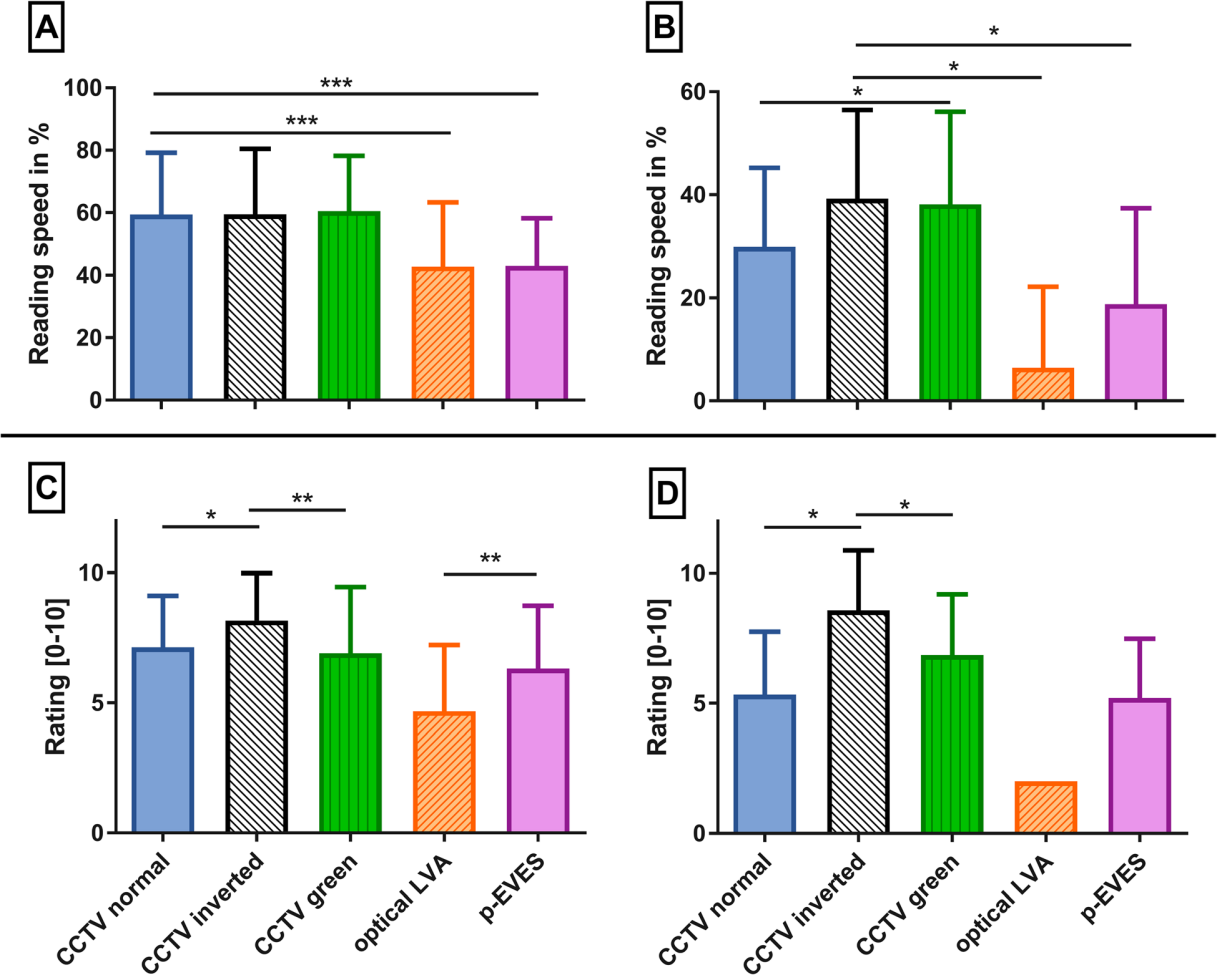
Reading performance with different LVAs of **(a)** all 34 patients and **(b)** only of 6 patients with severe visual impairment (BCVA≤20/400). Subjective rating of the different LVAs of **(c)** all 34 patients and **(d)** only of 6 patients with severe visual impairment. * = *p* < 0.05 ** = *p* < 0.01 *** = *p* < 0.001

Best reading performance for all patients could be achieved with CCTV (see Fig. 2a). Reading speed was significantly higher with CCTV and any contrast compared to optical LVAs and p-EVES (F2,76 = 24.1; *p* < 0.001, ANOVA). No significant differences were found between contrast settings. Normal reading speed (> 90%) could only be achieved by two patients with 2-fold magnification requirement. ‘No reading ability’ was scored as described before as 0 wpm to compare reading speed with and without LVAs [10]. With LVAs reading speed was significantly increased by 49% (*p* < 0.0001). Reading speed was also analyzed with patients allocated to two groups according to their grade of visual impairment (WHO 1–2 vs. WHO 3–4). The first group, comprised only of severely impaired (*n* = 4) and blind patients (*n* = 2), improved their reading speed with LVAs compared to best correction significantly to a lesser extent than the less afflicted group (32% vs. 59%, *p* = 0.04). Patients with severe VI showed significant differences in the analysis of varied contrast settings and LVAs (see Fig. 2b): Patients could read significantly faster if the background was black (no significant differences between white or green font color) compared to CCTV with normal contrast (*p* = 0.03) and again compared to optical LVAs and p-EVES (*p* < 0.001). The second group (WHO 3–4, *n* = 28) showed the same results as the analysis of all patients.

The patient-reported rating mostly resembled the results of the reading speed assessment (see Fig. 2c). However, most patients preferred reversed polarity to the normal (*p* = 0.02) and green-on-black contrast (*p* = 0.006). Interestingly, patients graded p-EVES significantly better than optical LVAs (*p* = 0.004). In the group of severely impaired patients reversed polarity was also rated significantly better compared to normal contrast (*p* = 0.02) with a higher mean difference between both (see Fig. 2d). The green-on-black contrast was rated slightly worse compared to reversed polarity, but better compared to normal contrast (both without significance). In patients with moderate VI there were no significant differences in ratings between the three contrast settings. Both portable LVAs were also significantly worse rated compared to CCTV (*p* = 0.01).

**Fig. 3:**
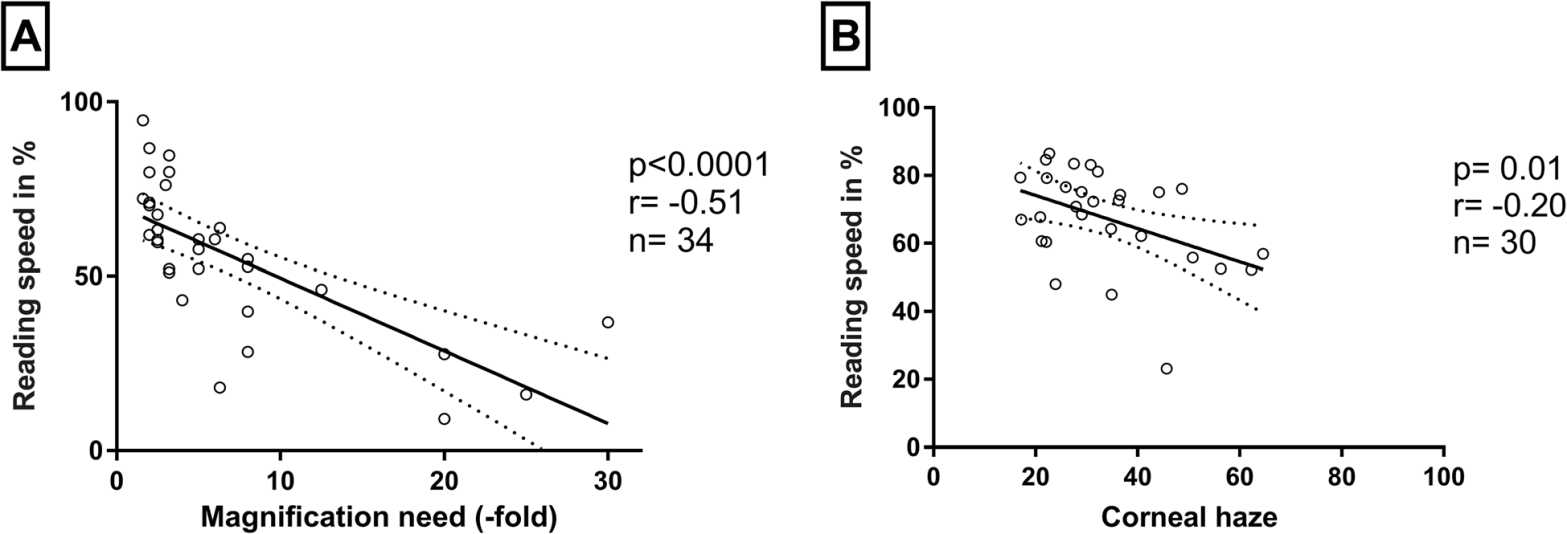
Reading performance was best predicted by **(a)** magnification need, but also significantly influenced by **(b)** the grade of corneal haze (0–100 Units, no haze – total opacification)

To evaluate which functional parameters influence the reading speed we performed linear regressions. The analysis revealed that reading speed is related to near visual acuity (F1,34 = 24.7; *p* = 0.001, *r* = 0.49) and magnification requirement (F1,34 = 23.1; *p* < 0.0001, *r* = − 0.51). Reading speed decreased with higher magnification and lower near visual acuity (s. Fig. 3a). Age, by contrast, showed no significant correlation (F1,34 = 0.7; *p* = 0.46, *r* = − 0.11). To further characterize the patient cohort, we also evaluated the correlation of reading speed to corneal haze. Linear regression showed a significant correlation (F1,34 = 6.8; p = 0.01, *r* = − 0.20). Higher opacification of the cornea lead to lower reading speed (s. Fig. 3b).

## Discussion

In this prospective, randomized cross-over clinical trial a detailed evaluation of patients with visual impairment due to corneal diseases and the best LVAs for them in terms of reading speed and patient-reported rating could be successfully accomplished. Our results show that visual rehabilitation of these specific patients can also be achieved with appropriately chosen LVAs and complement previous reports of treatment of retinal diseases [10, 11]. Whereas only six patients could read without a LVA, all patients could with the help of a LVA. This is similar to findings in AMD and confirms the great value of visual rehabilitation through the careful and adequate provision of LVAs [10]. Since reading is an integral part of many activities, the restoration of reading can also benefit other parts of daily living and consecutively independence and quality of life [11].

### Providing the appropriate LVA

Dependent on the grade of visual impairment, different LVAs could be shown to be effective in increasing the reading speed of the patients. This has previously been shown also for patients with retinal diseases [10, 11]. Hence, adequate visual rehabilitation needs the appropriate LVA: Severely afflicted patients (≥6-fold magnification requirement) could either not read at all with portable LVAs, or only poorly and needed a CCTV to restore their reading ability. In contrast, patients with low magnification requirement could read fluently with optical LVAs. This is in concordance with the visual rehabilitation of general low vision populations [19, 23, 38, 42] and emphasizes again the necessity to assess the magnification requirement before prescribing a LVA to ensure a cost-effective visual rehabilitation. Between both tested handheld LVAs there were no significant differences in reading speed, but patients rated p-EVES significantly better than optical magnifier. This is probably due to the fact that optical magnifier with higher magnification power restrict the field of vision distinctively. Besides that, p-EVES allow handling in habitual distance and better contrast [43]. Despite that, all patients could read fastest with CCTV. This was also the case in a recent trial by Jackson et al. (2017) with AMD patients, as well as in other prior trials with mainly retinal diseased patients [13, 24]. The superiority of the CCTV over portable LVAs is probably due to the wider field for viewing, which is especially beneficial with higher magnifications, since more words can be seen simultaneously [11, 44, 45]. In general, CCTVs seem ideal for leisure reading, since they offer binocular viewing in a habitual working distance and variable magnification and contrast settings. However, even with CCTVs available, patients’ expectations should be managed cautiously, because only two patients could achieve roughly the reading speed of a normal cohort even with CCTV. This has also been shown before for retinal diseases [10, 11]. Patients should thus be informed that LVAs cannot fully compensate a visual impairment in order to ensure their motivation [10]. Furthermore, CCTVs bind patients to a single location, and are more expensive which is why they can only be seen as an addition to a portable LVA for mobile use in shops, banks etc. [46]. Interestingly, already half of the patients used consumer electronics like smartphones as magnifying aid, because they are less stigmatizing than classical LVAs and provide the basic features of a p-EVES [17, 47]. Since many especially young patients own these devices anyway, they should be informed about the possibilities they comprise [17]. To conclude, the LVA for a patient should be chosen according to his magnification requirement, reading performance and personal preference. In principle, visual rehabilitation should aim for an optimal visual and economic outcome. Only after testing the possible LVAs, a patient can decide jointly with the low vision specialist, which device is appropriate for him. A limitation of our trial is that we could not evaluate the long-term use of each device. Future studies should consider this aspect and enable home phases and follow-up visits, since testing in the clinic is an artificial setting and only the daily use enables the patient to really test a LVA [24]. However, the most important task of a LVA is to provide adequate reading assistance, which we evaluated with standardized assessment of reading speed and personal rating.

### Contrast setting

Besides the choice of the right LVA – which we could show, is in patients with corneal diseases also dependent on the magnification requirement – the best contrast setting is of particular importance, since most of these patients suffer severely under glare sensitivity. In the analysis of our whole cohort, there were no differences between the contrast settings in terms of reading speed. This indicates that the most important factor is magnification. For further elucidation, we also analyzed two subgroups stratified according to their visual impairment grade. Whereas patients with moderate VI also showed, as expected, no differences between the contrast settings, this was not the case in patients with severe VI. Here, patients could read significantly faster with a black background compared to normal contrast. Previous studies already established this supremacy of reversed polarity over normal contrast for retinal diseases [22]. However, the color of the font had no significant influence in our cohort, although the mean reading speed was higher with reversed polarity compared to green on black contrast. In contrast, the patient reported rating showed a significant preference of the patients towards reversed polarity compared to normal and green-on-black contrast. This was the case in the analysis of the whole cohort as well. Hence, reversed polarity offers the fastest reading speed and best subjective rating in patients with severe visual impairment. This is in concordance with a previous trial by Legge et al. (1990), who showed that luminance contrast is superior to color contrast and that there is no additive interaction, since both are coded similar in our visual system [48]. When asked, many patients reported that the white background is glaring them. This discomfort is one probable explanation for the decreased reading speed. In less afflicted patients without severe glare, the contrast seems to be less important.

### Characterization of the patient group

Besides the evaluation of the visual rehabilitation, we also aimed to characterize the corneal diseased low vision patients. The reasons for visual impairments were diverse in contrast to regular visually impaired patients, who mostly suffer from AMD [11, 19, 49]. All patients had in common that they could not be treated with surgical procedures anymore and had to endure the consequences of visual impairment consecutively. Interestingly, many patients reported that they have not or only lately been informed about LVAs, since surgical options were still discussed. Even if there are possible options, in the future visual rehabilitation should not be postponed, to prevent a drastic decrease of quality of life and the incidence of depression. For a detailed characterization of these patients, we analyzed the visual impairment in regard to the cornea. For this, we measured the corneal haze with Pentacam. With the quantification of corneal haze and the analysis of its relationship with visual acuity, we could demonstrate that the vision of patients with corneal opacifications depends mainly on the cornea. Previous publications already established the corneal densitometry as a tool to quantify the disease stage [31–35]. In contrast to our results, Kamiya et al. (2017) found no significant correlation between corneal densitometry and visual acuity in patients with band keratopathy, which might be a result of the nature of the specific disease, since the visual axis is not necessarily occluded [50]. For further elucidation of the factors determining the visual acuity of these patients, we performed a multiple regression with corneal haze, age and LIVA. It showed a higher correlation, which indicates that the age-dependent capability of the retina is also important in addition to the grade of corneal haze. Following the decreased visual acuity with higher opacification, reading speed decreased as well with increased corneal haze, as evaluated by linear regression. Mathews et al. (2017) has been able to show before that with higher corneal staining the reading speed decreases [51]. Our results with an objective method confirmed these findings.

### Prediction of performance

For provision of the appropriate LVA for a visually impaired patient it is important to predict how well the patient will perform with different LVAs [24]. Therefore, we evaluated the reading speed concerning morphological and functional examinations. With linear regression, we could show that reading speed decreases with higher magnification requirement and lower near visual acuity. The influence of visual acuity on reading performance has been shown prior to this for AMD patients [10]. Furthermore, we could show that the objective parameter corneal haze has as well a significant impact on the reading speed. This is in concordance with our finding of decreased visual acuity with worse corneal opacification. However, corneal haze alone is not a valid parameter for precise prediction of VA or reading speed, because of a high variability. Hence, visual rehabilitation for corneal patients can also rely on measurement of the magnification requirement and/or on near visual acuity as in patients with retinal diseases.

## Conclusions

Reading ability in patients with corneal diseases can be restored successfully and improved with LVAs. The appropriate LVA is dependent on the grade of visual impairment, assessed as magnification requirement or near visual acuity. Highest reading speeds could be achieved with CCTVs, especially with black background, probably due to less glare. Severely afflicted patients rated reversed polarity with a CCTV best, less afflicted patients could also improve their reading speed sufficiently with portable LVAs. Quantification of corneal haze can assist to estimate visual acuity and reading performance.

## Data Availability

The datasets used and analysed during the current study are available from the corresponding author on reasonable request.

## Abbreviations

AMD: Age-related macular degeneration
BCVA: Best correct visual acuity
CCTV: Closed circuit television; also electronic desk video magnifiers
DALK: Deep anterior lamellar keratoplasty
DMEK: Descemet membrane endothelial keratoplasty
ETDRS: Early treatment of diabetic retinopathy study
GAT: Goldmann applanation tonometry
GSU: Grayscale units
GvHD: Graft versus host disease
IReST: International-reading-speed-texts
IOP: Intraocular pressure
LIVA: Laser interference visual acuity measurement
logMAR: Logarithmic value of the minimum angle of resolution
LVA: Low vision aid
PK: Perforating keratoplasty
p-EVES: Portable electronic vision enhancement systems
SD-OCT: Spectral-domain optical coherence tomography
VA: Visual acuity
VI: Visual impairment
WHO: World Health Organization
Wpm: Words per minute
